# Turnover intention and the moderating role of career shocks an empirical study of medical staff in public hospitals in Guangzhou, China

**DOI:** 10.3389/fpsyg.2025.1567583

**Published:** 2025-09-05

**Authors:** Fan Yang, Henrique Duarte, Jianping Chen, Chenxi Zu

**Affiliations:** ^1^The Second Affiliated Hospital, Guangzhou Medical University, Guangzhou, China; ^2^Iscte Business School, Instituto Universitário de Lisboa (ISCTE-IUL), Lisbon, Portugal; ^3^Business Research Unit (BRU-IUL), Instituto Universitário de Lisboa (ISCTE-IUL), Lisbon, Portugal; ^4^Guizhou Cancer Hospital, Guiyang, China; ^5^The Third Affiliated Hospital, Sun Yat-sen University, Guangzhou, China

**Keywords:** turnover intention, career shocks, leader-member exchange, job embeddedness, job satisfaction, public hospitals, medical staff

## Abstract

**Objective:**

This study aims to explore how Leader-Member Exchange (LMX), Job Embeddedness (JE), Job Satisfaction (JS), and Career shocks (CS) affect hospital staff’s turnover intention. By offering theoretical and practical insights, it reassures the reader of the study’s value in curbing employee departures and building a more stable workforce.

**Methods:**

Using a longitudinal design and self-administered questionnaires, data were collected in two rounds from 500 medical staff in 12 tertiary public hospitals in Guangzhou, China, between May and September 2023. The data were analyzed with SPSS 26 and PROCESS macro for SPSS 4.1, including descriptive statistics, correlation analysis, and mediation and moderation analyses.

**Results:**

LMX, JE, and JS were significantly negatively correlated with Turnover Intention (TI) (*r* = –0.365, *p* < 0.01; *r* = –0.571, *p* < 0.01; *r* = –0.517, *p* < 0.01). JS fully mediated the link between LMX and TI (ab = –0.384, 95%CI: [–0.497, –0.285]) and partially mediated the link between JE and TI (ab = –0.237, 95%CI: [–0.350, –0.123]). CS significantly moderates these relationships: positive CS enhanced employees’ JE and JS, while negative CS weakened organizational commitment and increased turnover likelihood.

**Discussion:**

CS can temporarily intensify the relationships between LMX, JE, JS, and TI under certain circumstances, likely due to heightened employee sensitivity to leadership support, work environment, and career development. This moderation effect also highlights that employees’ demand for organizational support increases when facing career challenges, and high-quality LMX and JE can provide essential assistance for employees to navigate career shocks.

**Conclusion:**

By investigating the impact of LMX, JE, JS, and CS on turnover intention, this study offers a fresh perspective on the occupational behavior of medical staff in public hospitals. The findings not only enrich LMX and career shocks theories but also provide practical guidance for managers. This guidance empowers managers to reduce employee turnover intention, making them feel capable and ready to implement these strategies. As a primary goal, the abstract should render the general significance and conceptual advance of the work clearly accessible to a broad readership. References should not be cited in the abstract. Leave the Abstract empty if your article does not require one – please see the “Article types” on every Frontiers journal page for full details.

## 1 Introduction

Public hospitals form the backbone of China’s healthcare system. They are crucial for enhancing the equity and accessibility of essential medical services. Moreover, they play a key role in preventing and controlling major epidemics like COVID-19 and safeguarding people’s lives and health (National Health Commission, 2021). Hospital medical staff, essential for service provision, research, and education, ensure the hospitals’ ongoing high-quality care. However, multiple studies have shown that medical staff in Chinese public hospitals have a high intention and rate of turnover (Chen, 2021; Wang, 2021; Wu et al., 2021; YIMI Research, 2022). High turnover intentions and rates affect public hospitals’ operational efficiency and service quality and restrict the development of medical and health causes.

Turnover intention refers to an employee’s thoughts or tendencies to leave their current job or organization within a specific period. Turnover intention is often seen as an effective indicator for predicting actual turnover behavior (Hom and Griffeth, 1995). Previous studies often used variables of employees’ subjective attitudes for analysis, such as job satisfaction, organizational identification, and organizational commitment. However, research has shown that traditional attitude variables have limited explanatory power for employees’ turnover tendencies (Griffeth et al., 2000; Hom and Griffeth, 1995; Maertz and Campion, 2004). Therefore, it is necessary to explore new variables beyond traditional attitudinal variables to investigate employee turnover tendencies.

This study has two main objectives. First, it evaluates how Leader-Member Exchange (LMX), Job Embeddedness (JE), and Job Satisfaction (JS) impact employee turnover intention. Second, it explores the moderating role of career shocks in the relationships between JE, LMX, JS, and turnover intention. By pinpointing key drivers and potential moderating mechanisms, the study aims to offer practical HR strategies for hospital management to reduce medical staff turnover, equipping them with the necessary tools to address this critical issue.

Previous studies have extensively examined factors influencing turnover intention (Hom et al., 2017), but there are gaps in understanding the complex interplay between Leader-Member Exchange (LMX), Job Embeddedness (JE), Job Satisfaction (JS), and turnover intention in the context of public hospitals in China. Existing research has often focused on transformational (Deng, 2023; Dong et al., 2024), with less attention paid to career shocks and their potential moderating role (Feng et al., 2021; Huang and Cui, 2021). This study addresses this gap by integrating LMX, JE, JS, and turnover intention into a unified framework and examining the moderating effect of career shocks. It provides a more comprehensive perspective on factors influencing medical staff turnover intention in public hospitals and offers practical guidance for hospital managers to develop targeted retention strategies.

## 2 Literature review

Job embeddedness reflects the strong ties and interdependence between individuals and their work environment, forming a powerful force for retention that increases the resistance and costs for individuals when considering leaving their jobs (Mitchell et al., 2001). Setthakorn et al. (2024) further confirmed the relationship between job embeddedness and turnover intentions in their study in Thailand and Indonesia. Highly embedded employees have established close connections and ties within their work or organization, increasing the cost and difficulty of leaving, leading us to propose the hypothesis:

*H1*: Job Embeddedness (JE) is negatively correlated with Turnover Intention (TI).

The Leader-Member Exchange (LMX) theory describes the social exchanges between leaders and subordinates based on specific relationships (Graen and Uhl-Bien, 1995). Due to leaders’ limited time and energy, they inevitably differentiate among their subordinates, building various types of relationships. High-quality LMX relationships can improve employee job satisfaction and performance, reduce work stress, increase communication frequency, and enhance work efficiency and team outcomes. Conversely, low-quality LMX relationships may lead to poor job performance, higher stress, dissatisfaction, and increased turnover intentions. leading us to propose the hypothesis:

*H2*: There is a negative correlation between LMX and turnover intentions.

Job satisfaction is an essential indicator for measuring employees’ psychological health and quality of life and a key factor affecting organizational stability and employees’ intentions to leave. This multidimensional concept has received extensive attention and research in organizational behavior and human resource management (Judge et al., 2017). With in-depth Research, scholars have found a close association between job satisfaction and turnover intentions. When employees are satisfied with work content, working environment, and compensation, their sense of belonging and loyalty to the organization will be enhanced, reducing the intention to leave (Jackofsky and Peters, 1983). Furthermore, much empirical research supports this view, proving that job satisfaction is a valid indicator for predicting employee turnover intentions (Mobley et al., 1978). Making us to propose the following hypothesis

*H3*: There is a negative relation between Job satisfaction (JS) and turnover intention (TI).

When discussing the turnover intentions of medical staff, job satisfaction plays an essential role. LMX and Job Embeddedness (JE) influence employee job satisfaction. The LMX theory suggests that high-quality relationships between leaders and subordinates can enhance subordinates’ job satisfaction, as these relationships are usually accompanied by trust, respect, and support (Graen and Uhl-Bien, 1995). Gerstner and Day (1997) research indicates that LMX indirectly affects turnover behavior by influencing employees’ job satisfaction and other attitudes. On the other hand, the job embeddedness theory emphasizes the connections between employees and their work, organization, and community, increasing employees’ motivation to stay in their current positions and thereby improving job satisfaction (Mitchell et al., 2001). When employees feel support from leaders and connections with colleagues, they are more likely to be satisfied with their jobs, and this satisfaction, in turn, reduces their willingness to leave the organization.

LMX and JE indirectly reduce turnover intentions by increasing job satisfaction. Thus, we hypothesize:

*H4a*: Job satisfaction mediates the relationship between LMX and turnover intentions.

*H4b*: Job satisfaction mediates the relationship between Job Embeddedness and turnover intentions.

Career shocks, derived from the “systemic shock” in the Unfolding Model of Voluntary Employee Turnover, refers to destructive and unique events that occur beyond an individual’s control, triggering a process of careful reflection on one’s career (Akkermans et al., 2018). Its effect encourages individuals to reassess their career development paths and directions (Seibert et al., 2013). These unusual events have a profound impact on the careers of medical professionals. They can stimulate deep reflection on one’s current career situation and may alter career decisions (Feng et al., 2021). Such events can be categorized into positive career shock (e.g., unexpected promotions, receiving significant awards) and negative career shocks (e.g., overlooked promotions, project failures). Positive career shocks enhance employees’ identification with and sense of belonging to their current careers, elevate their status and reputation within organizations and professional fields, and not only bring a sense of achievement and satisfaction but potentially inspire tremendous work enthusiasm and career motivation (Kraimer et al., 2019). These positive events occurring within organizations are akin to placing “golden handcuffs” on employees (Seibert et al., 2016), strengthening their connection to the organization and reducing their willingness to leave. However, special positive shocks related to career opportunities, such as unexpected job offers, may inspire employees to explore more career possibilities, potentially leading to thoughts of changing organizations, industries, or positions and even resulting in boomerang behavior (Amarneh et al., 2021; Lee et al., 2008). In contrast, negative career shocks often trigger dissatisfaction and disappointment with one’s current career and may even lead to the desire to leave. These events can negatively impact an individual’s career mindset and planning. Additionally, negative political events and significant organizational changes, such as layoffs, mergers and acquisitions, or ethical scandals, can also affect employees, threatening their career stability and prospects. Personal life events, such as a spouse needing to change cities for a new job, divorce, serious illness, or death of a family member (Seibert et al., 2016), can similarly have a profound impact on an individual’s career status and decisions, forcing them to re-examine and adjust their career plans and goals.

The leader-member exchange (LMX) theory emphasizes that the relationship quality between leaders and subordinates affects employee behavior and attitudes (Graen and Uhl-Bien, 1995). This underscores the crucial role of leaders in supporting their subordinates, particularly during challenging times such as career shocks. Employees facing such shocks may rely more on their leaders’ support and resources to deal with challenges, affecting their behavior and attitudes. For instance, when medical staff feel frustrated due to failed promotions or research project applications, these negative career shocks may weaken their commitment to the hospital, reduce the quality of LMX, and thus increase the likelihood of leaving. These events may undermine the medical staff’s trust in and commitment to the organization, lowering the quality of LMX. In such cases, the negative impact of high-quality LMX on the intention to leave may be weakened. Negative career shocks may lead medical staff to reassess their career development paths, increasing the likelihood of leaving. From the perspective of job embeddedness, Mitchell et al. (2001) believe that the close connection and interdependence between employees and their work environment can influence their intention to leave. When medical staff experience positive career shocks, such as unexpected promotions or recognition, it enhances their professional identity and sense of belonging, thereby increasing their job embeddedness. In such cases, the negative impact of job embeddedness on the intention to leave may be further strengthened. This is because positive career shocks improve job satisfaction among medical staff and strengthen their connection with the organization, making them more likely to stay in their current positions.

Based on the above discussion, we posit that career shocks may moderate the effects of leader-member exchange (LMX), job embeddedness, and job satisfaction on turnover intention. Specifically, we hypothesize that:

*H5a*: Career shocks may intensify the negative relationship between LMX and turnover intentions, which is more pronounced when career shocks are high.

*H5b*: Career shocks may intensify the negative relationship between job embeddedness and turnover intentions, which is more pronounced when career shocks are high.

*H5c*: Career shocks may intensify the negative relationship between job satisfaction and turnover intentions, which is more pronounced when career shocks are high.

**FIGURE 1:**
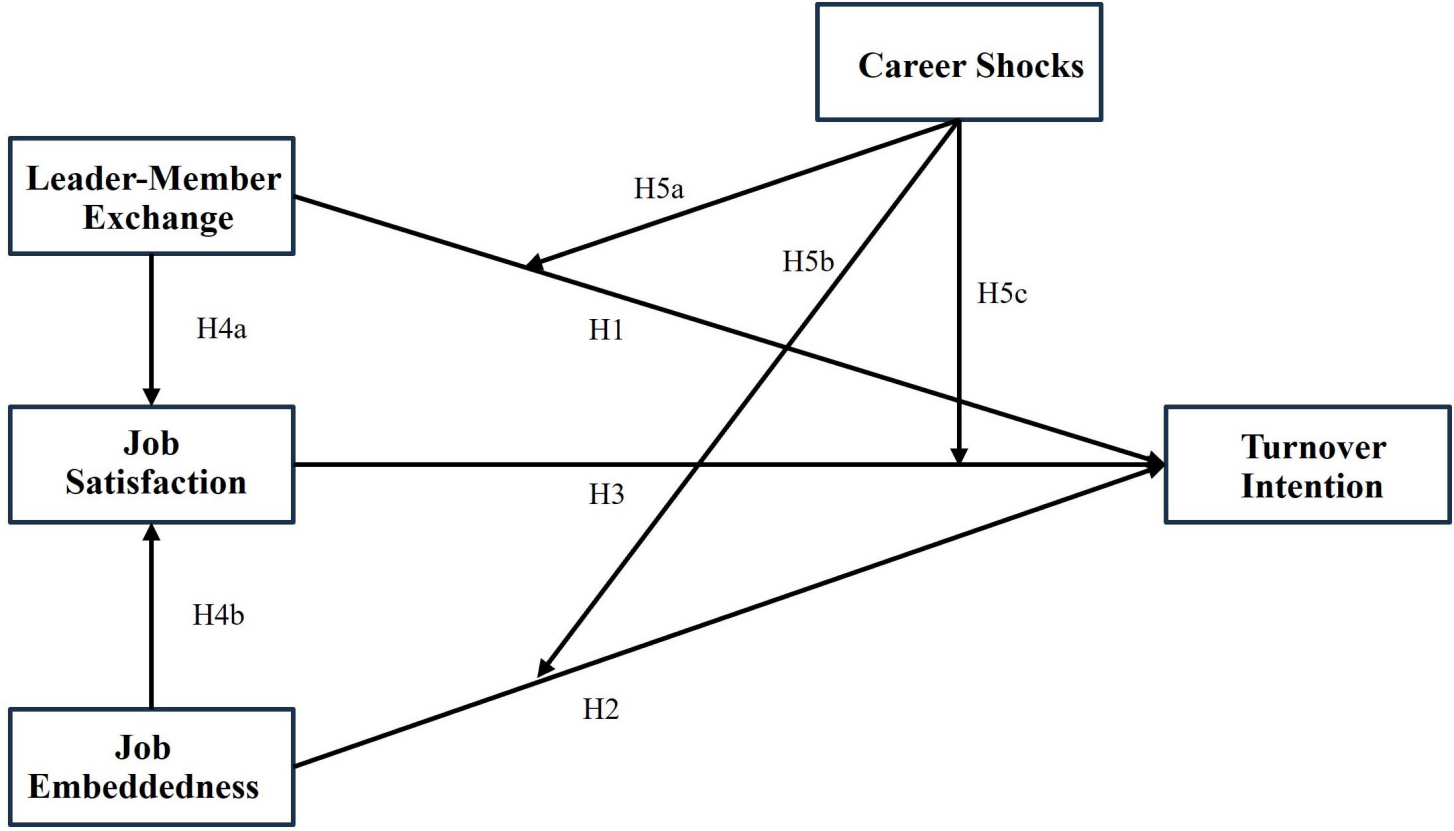
Framework diagram.

Previous research has shown that leader-member exchange (LMX), job embeddedness (JE), and job satisfaction (JS) can influence medical professionals’ turnover intention (TI). However, most studies have focused on analyzing these factors individually, with little exploration of their interactions. Moreover, the role of career shocks (CS) as a potential moderator in medical professionals’ turnover intention has not been thoroughly studied. This study aims to fill this gap by constructing an integrated framework to systematically explore the complex relationships among these variables and verify the moderating effect of career shocks (see Figure 1). The practical implication of this research is significant, as it will provide public hospital managers with a more comprehensive theoretical basis and practical guidance for developing effective human resource management strategies to reduce turnover rates.

## 3 Materials and methods

### 3.1 Research design and participants

This study employed longitudinal research design and collected data using self-administered questionnaires. The research adopted a multi-stage random sampling method. From May to September 2023, the research team conducted two rounds of questionnaire surveys, each involving 500 participants. As of April 26, 2023, there were 20 tertiary public hospitals in Guangzhou, China, including 11 general and nine specialized hospitals. For this study, seven general hospitals and five specialized hospitals were randomly selected, totaling 12 tertiary public hospitals. These hospitals were chosen because they are located in different administrative districts of Guangzhou and cover various types of medical service institutions. Moreover, they provide comprehensive health services and have high medical care, teaching, and research standards, making them suitable for examining the turnover rates of medical professionals in Guangzhou, China. Finally, at least 40 medical staff members were randomly selected from each chosen hospital using a random number table method.

All study procedures were conducted with the highest ethical standards and were approved by the Ethics Committee of the Second Affiliated Hospital of Guangzhou Medical University (approval number: 2023-ks-07). This approval underscores the rigorous ethical review process the study underwent. Furthermore, all participants were fully informed of the study’s purpose and provided signed consent forms, ensuring their rights and privacy were respected throughout the research process. The inclusion criteria for participants were as follows: participants must (1) have a formal labor contract with the hospital; (2) have worked at the hospital for at least 1 year; (3) have signed an informed consent form and voluntarily completed the questionnaire. Trained volunteers created electronic versions of the scales and generated corresponding QR codes using a professional online survey website.^1^ Respondents could easily access and fill out the online questionnaire by scanning the QR code with their smartphones. Volunteers explained the purpose and principles of the study to the respondents, guided them on how to fill out the questionnaire, and obtained their informed consent. To protect the respondents’ privacy and match the results of the two rounds of questionnaire surveys, we designed a unique identification code, which was created by combining part of the respondent’s surname and part of their mobile phone number. A total of 338 valid questionnaires were successfully matched in the two rounds of surveys.

Before testing our hypotheses, we evaluated the psychometric quality of the scales used in this study through exploratory factor analysis (EFA). Based on the results, we took a rigorous approach and eliminated items with low factor loadings, ensuring the most accurate measurement of the variables. Additionally, we conducted confirmatory factor analysis (CFA) to assess the association between the individual items in the measurement model and the concepts they represent, thereby enhancing the accuracy and reliability of the study. The results indicated that the scales fit the data reasonably well (χ^2^ = 1072.589, *p* < 0.01, χ^2^/*df* = 2.56, CFI = 0.918, IFI = 0.918, TLI = 0.909, GFI = 0.827, RMSEA = 0.068, LO = 0.063, HI = 0.073). We also assessed the discriminant validity of the scales using average variance extracted (AVE) and composite reliability (CR). The results showed that the AVE values for almost all scales were above the reference value of 0.50, and the CR values were above 0.70. After ensuring good reliability and acceptable discriminant validity within each scale, we performed multiple linear regression on the sample to test the main hypotheses.

### 3.2 Sociodemographic data and work characteristics

Data on respondents’ gender, age, marital status, years of work experience, education level, monthly income, LMX, job embeddedness, job satisfaction, turnover intention, and career shocks were collected using a structured questionnaire.

### 3.3 Measurement of turnover intention

The Turnover Intention Scale developed by Mobley et al. (1979) was selected for use in this study. This scale consists of 4 items, measured using a 5-point Likert scale, ranging from 1 (strongly disagree) to 5 (strongly agree). The higher the total score, the stronger the respondent’s intention to leave their job. The Cronbach’s α coefficient is 0.755.

### 3.4 Measurement of LMX

The LMX scale developed by Wang et al. (2005) was utilized for this measurement. This scale comprises seven items, rated on a 5-point Likert scale ranging from 1 (strongly disagree) to 5 (strongly agree). A higher total score indicates a closer relationship between the respondent and their leader. The Cronbach’s α coefficient is 0.92.

### 3.5 Measurement of job embeddedness

The Job Embeddedness scale developed by Crossley et al. (2007) was employed for this assessment. This scale includes seven items, rated on a 5-point Likert scale, ranging from 1 (strongly disagree) to 5 (strongly agree). A higher total score indicates a deeper level of job embeddedness for the respondent. The Cronbach’s α coefficient is 0.862.

### 3.6 Measurement of job satisfaction

The Overall Job Satisfaction scale developed by Tsui et al. (1992) was used for this measurement. This scale includes six items, rated on a 5-point Likert scale, ranging from 1 (strongly disagree) to 5 (strongly agree). A higher total score indicates a higher level of job satisfaction among the respondents. The Cronbach’s α coefficient is 0.75.

### 3.7 Measurement of career shocks

The Career shocks Event scale developed by Ali et al. (2020) for the Chinese context was used for this measurement. With nine items, this scale categorizes career shocks events into two significant types: Positive Career shocks (PCS) events and Negative Career shocks (NCS) events. It uses a 5-point Likert scale, ranging from 1 (strongly disagree) to 5 (strongly agree). A higher total score indicates a greater level of career shocks experienced by the respondent. The Cronbach’s α coefficient for the Positive Career Shocks (PCS) subscale is 0.92, and for the Negative Career Shocks (NCS) subscale, it is 0.94.

### 3.8 Statistical analysis

Data preprocessing and analysis were conducted using SPSS 26 and the PROCESS macro for SPSS 4.1. SPSS was utilized for descriptive statistical, correlation, and exploratory factor analyses. The PROCESS macro was employed to analyze mediating effects and moderating effects. In the analysis, we adjusted for covariates that might affect turnover intention and used the Bootstrap method to enhance the robustness of the results. You may insert up to 5 heading levels into your manuscript as can be seen in “Styles” tab of this template. These formatting styles are meant as a guide, as long as the heading levels are clear, Frontiers style will be applied during typesetting.

## 4 Results

### 4.1 Common method biases analyses

To control for common method bias, Harman’s single-factor test was performed on the scales (Fang et al., 2020; Podsakoff et al., 2003). After principal component analysis, six factors with eigenvalues greater than one were extracted. The first factor accounted for only 39.28% of the variance, below the 50.0% threshold (Podsakoff et al., 2003). Thus, common method bias is not a serious concern in this study.

### 4.2 Preliminary analyses

Table 1 provides a comprehensive overview of the 338 respondents, highlighting their demographic and job-related features. Predominantly female (72.2%) and highly educated, with 90.5% holding a bachelor’s degree or higher. The majority were married (73.1%), and doctors and nurses represented approximately half of the sample. A significant proportion (67.2%) held mid-level or higher professional titles, and the majority (78.1%) had over 6 years of work experience.

**TABLE 1:** Comparison of turnover intention among medical staff of different demographic characteristics (*N* = 338).

Variable	N(%)	Turnover intention (M ± SD)	*F*	*P*
Gender			2.143	0.144
Male	94(27.8%)	1.904 ± 0.842		
Female	244(72.2%)	1.897 ± 0.935		
Age (years)			6.541	0.000
29 or below	74(21.9%)	2.189 ± 2.189		
30–39	140(41.4%)	1.930 ± 1.930		
40–49	95(28.1%)	1.784 ± 1.784		
50 or above	29(8.6%)	1.379 ± 1.379		
Education background			1.064	0.365
College or below	32(9.5%)	2.031 ± 0.960		
Bachelor	170(50.3%)	1.952 ± 0.983		
Master	103(30.5%)	1.774 ± 0.794		
Doctor	33(9.8%)	1.886 ± 0.786		
Marital status			7.119	0.001
Married	247(73.1%)	1.792 ± 0.869		
Unmarried	85(25.1%)	2.215 ± 0.964		
Others (divorce)	6(1.8%)	1.833 ± 0.801		
Personnel type			10.679	0.001
Doctor	166(49.1%)	1.798 ± 1.798		
Nurse	172(50.9%)	1.996 ± 1.996		
Professional title			2.914	0.022
Primary	110(32.5%)	2.132 ± 1.014		
Intermediate	135(39.9%)	1.824 ± 0.869		
Vice senior	70(20.7%)	1.732 ± 0.778		
Senior	22(6.5%)	1.716 ± 0.821		
Ungraded	1(0.3%)	2.000 ± 0.000		
Length of service			7.369	0.000
5 years or below	74(21.9%)	2.041 ± 0.968		
6–15 Years	152(45%)	1.990 ± 0.932		
16–25 years	73(21.6%)	1.890 ± 0.846		
26 years or above	39(11.5%)	1.288 ± 0.515		
Monthly income			2.193	0.089
Less than 10,000	105(31.1%)	2.062 ± 1.006		
10,001–20,000	163(48.2%)	1.865 ± 0.885		
20,001–30,000	59(17.5%)	1.767 ± 0.807		
30,000 or above	11(3.3%)	1.546 ± 0.579		

Correlation analyses (Table 2) revealed that turnover intention was significantly and negatively correlated with leader-member exchange (*r* = –0.365, *p* < 0.01), job embeddedness (*r* = –0.571, *p* < 0.01), and job satisfaction (*r* = –0.517, *p* < 0.01), and significantly and positively correlated with negative career shocks (*r* = 0.247, *p* < 0.01). These findings suggest that a strong leader-member relationship, job satisfaction, and job embeddedness can potentially reduce turnover intention, while negative career shocks can increase it. Additionally, leader-member exchange was positively correlated with job embeddedness and job satisfaction, while job embeddedness was positively correlated with job satisfaction but negatively correlated with negative career shocks. Job satisfaction was positively associated with positive career shocks and negatively with negative career shocks. Positive and negative career shocks correlated positively (*r* = 0.441, *p* < 0.01).

**TABLE 2:** Descriptive statistics and correlations among the key variables.

Variable	*Mean*	*SD*	1	2	3	4	5	6	7	8	9	10	11
1.Gender	1.720	0.449	−										
2.Age	2.230	0.889	-0.127*	−									
3.Education background	2.410	0.792	-0.241**	0.223**	−								
4.Length of service	2.230	0.920	0.003	0.834**	0.011	−							
5.TI (t1)	1.899	0.909	-0.004	-0.229**	-0.074	-0.208**	−						
6.TI (t2)	1.902	0.964	-0.005	-.205**	0.005	-0.193**	0.483**	−					
7.LMX (t1)	3.927	0.729	0.063	-0.063	-0.185**	0.062	-0.365**	-0.294**	−				
8.JE (t1)	3.867	0.776	0.019	0.219**	-0.043	0.239**	-0.571**	-0.446**	0.542**	−			
9.JS (t1)	3.821	0.774	0.124*	0.014	-0.095	0.079	-0.517**	-0.419**	0.654**	0.738**	−		
10.JS (t2)	3.919	0.769	0.062	0.051	-0.229**	0.086	-0.404**	-0.409**	0.477**	0.551**	0.653**	−	
11.PCS (t2)	2.946	1.088	-0.019	-0.169**	-0.076	-0.144**	0.051	0.116*	0.036	0.033	0*	0.148**	-
12.NCS (t2)	2.932	1.091	-0.06	-0.081	0.06	-0.105	0.282**	0.306**	-0.191**	-0.195**	-0.230**	-0.189**	0.441**

*N* = 338.

*p < 0.05,

**p < 0.01; TI, turnover intention; LMX, leader-member exchange; JE, job embeddedness; JS, job satisfaction; PCS, positive career shock; NCS, negative career shock. *t* = measurement wave.

### 4.3 Mediating analyses

The results (see Table 3) showed that after controlling gender, age, and length of service, leader-member exchange (LMX) was significantly and negatively associated with turnover intention (TI) (*B* = –0.487, *p* < 0.001). When job satisfaction (JS) was introduced as a mediating variable, the direct effect of LMX on TI was no longer significant (*B* = 0.322, *p* > 0.05), while JS was significantly and negatively related to TI (*B* = –0.550, *p* < 0.001). LMX was a significant predictor of JS (*B* = 0.699, *p* < 0.001). The bias-corrected percentile bootstrapping analysis indicated that the mediating effect of JS in the relationship between LMX and TI was significant (ab = –0.384, 95% CI: –0.497, –0.285), accounting for 78.84% of the total effect. This suggests that JS fully mediated the relationship between LMX and TI.

**TABLE 3:** Testing Job satisfaction as a mediator in the relationship between leader-member exchange, job embeddedness and turnover intention.

Model pathways	*R*2	*B*	SE	β	t	95% CI
**Job satisfaction as a mediator in the relationship between LMX and turnover intention**						
Leader-member exchange →Turnover intention	0.200	-0.487	0.063	-0.391	-7.784	(–0.610, –0.364)
Leader-member exchange →Job satisfaction	0.440	0.699	0.045	0.659	15.683	(0.611, 0.787)
Leader-member exchange →Turnover intention	0.322	-0.1031	0.0761	-0.083	-1.355	(–0.253, 0.047)
Job satisfaction →Turnover intention		-0.550	0.071	-0.468	-7.748	(–0.689, –0.41)
Indirect effect		-0.384	0.054			(–0.497, –0.285)
Direct effect		-0.103	0.076			(–0.253, 0.047)
Total effect		-0.487	0.063			(–0.610, –0.364)
**Job satisfaction as a mediator in the relationship between job embeddedness and turnover intention**						
Job embeddedness →turnover intention	0.339	-0.644	0.054	-0.550	-11.967	(–0.750, –0.538)
Job embeddedness →job satisfaction	0.576	0.763	0.037	0.765	20.788	(0.690, 0.835)
Job embeddedness →turnover intention	0.368	-0.408	0.080	-0.348	-5.105	(–0.565, –0.251)
Job satisfaction →turnover intention		-0.310	0.079	-0.264	-3.939	(–0.465, –0.155)
Indirect effect		-0.237	0.058			(–0.350, –0.123)
Direct effect		-0.408	0.080			(–0.565, –0.251)
Total effect		-0.644	0.054			(–0.750, –0.538)

Covariates used to control including gender, age, education level, and length of service. B, regression coefficient; SE, standard error.

Our results, as shown in Table 3, revealed a significant and negative association between job embeddedness (JE) and turnover intention (TI) (*B* = –0.644, *p* < 0.001), even after controlling relevant variables. When job satisfaction (JS) was introduced as a mediating variable, we found that both JE (*B* = –0.408, *p* > 0.05) and JS (*B* = –0.310, *p* < 0.001) were negatively related to TI. Importantly, JE was a significant predictor of JS (*B* = 0.763, *p* < 0.001). Our bias-corrected percentile bootstrapping analysis confirmed the significant mediating effect of JS in the JE-TI relationship (ab = –0.237, 95% CI: –0.350, –0.123), accounting for 36.71% of the total effect. This suggests that JS acts as a partial mediator in the JE-TI relationship, highlighting the complex dynamics of play.

### 4.4 Moderated mediation analyses

In summary, the moderated mediation analyses (see Table 4) showed that leader-member exchange (LMX) and job satisfaction (JS) were negatively related to turnover intention (TI) (*B* = –0.160, *p* < 0.05; *B* = –0.453, *p* < 0.001), whereas positive career shocks (PCS) were positively associated with TI (*B* = 0.101, *p* < 0.05). The interaction between LMX and PCS was significant (*B* = –0.173, *p* < 0.01), indicating that PCS moderated the relationship between LMX and TI. Similarly, the interaction between JS and PCS was significant (*B* = 0.225, *p* < 0.001), indicating that PCS moderated the relationship between JS and TI.

**TABLE 4:** Testing career shocks as a moderator.

Model pathways	R2	*B*	SE	t	95% CI
Leader-member exchange →job satisfaction	0.237	0.513	0.052	9.912	(0.411, 0.615)
Leader-member exchange →turnover intention	0.275	-0.160	0.073	-2.190	(–0.303 −0.016)
Job satisfaction →turnover intention		-0.453	0.069	-6.570	(–0.588 −0.317)
Positive career shock →turnover intention		0.101	0.045	2.261	(0.013, 0.188)
LMX × Positive career shock →Turnover intention		-0.173	0.062	-2.783	(–0.296 −0.051)
Job satisfaction × positive career shock →turnover intention		0.225	0.058	3.888	(0.111, 0.339)
Job embeddedness →job satisfaction	0.310	0.559	0.047	12.005	(0.467, 0.650)
Job embeddedness →turnover intention		-0.331	0.070	-4.699	(–0.469 −0.192)
Job satisfaction →turnover intention		-0.343	0.070	-4.885	(–0.481 −0.205)
Positive career shock →turnover intention		0.101	0.043	2.327	(0.016, 0.186)
Job embeddedness × positive career shock →turnover intention		-0.162	0.057	-2.846	(–0.275 −0.050)
Job satisfaction × Positive career shock →Turnover intention		0.237	0.059	4.043	(0.122, 0.353)
Leader-member exchange →job satisfaction	0.237	0.513	0.052	9.912	(0.411, 0.615)
Leader-member exchange →turnover intention	0.292	-0.182	0.072	-2.518	(–0.324 −0.040)
Job satisfaction →turnover intention		-0.376	0.067	-5.613	(–0.508 −0.244)
Negative career shock →turnover intention		0.148	0.047	3.161	(0.056, 0.240)
LMX × Negative career shock→turnover intention		-0.181	0.060	-3.019	(–0.298 −0.063)
Job satisfaction × negative career shock →turnover intention		0.189	0.058	3.274	(0.076, 0.302)
Job embeddedness →job satisfaction	0.310	0.559	0.047	12.005	(0.467, 0.650)
Job embeddedness →turnover intention	0.322	-0.316	0.070	-4.523	(–0.45 −0.179)
Job satisfaction →turnover intention		-0.292	0.069	-4.240	(–0.428 −0.157)
Negative career shock →turnover intention		0.153	0.046	3.331	(0.063, 0.243)
Job embeddedness × negative career shock →turnover intention		-0.184	0.061	-3.040	(–0.304 −0.065)
Job satisfaction × negative career shock →turnover intention		0.196	0.058	3.376	(0.082, 0.311)

Covariates used to control including gender, age, education level, and length of service. B, regression coefficient; SE, standard error.

**FIGURE 2:**
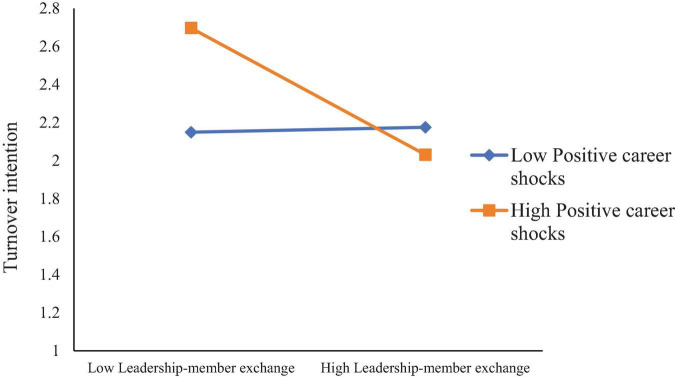
Moderation of positive career shocks to leadership-member exchange and turnover intention.

**FIGURE 3:**
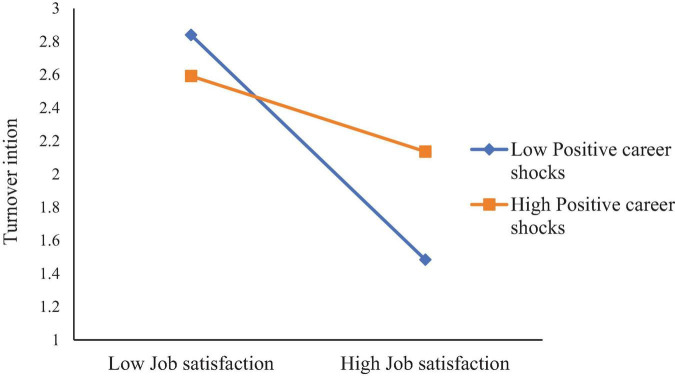
Moderation of positive career shocks to job satisfaction and turnover intention.

Simple slope tests (see Figure 2) revealed that when PCS was high, LMX was negatively related to TI (B_simple_ = –0.3481, *p* < 0.001), but this relationship was not significant when PCS was low (B_simple_ = 0.0288, n.s.). Likewise, when PCS was high (see Figure 3), JS was negatively related to TI (B_simple_ = –0.2072, *p* < 0.001), whereas when PCS was low, JS was more strongly negatively related to TI (B_simple_ = –0.6978, *p* < 0.001).

Additionally, job embeddedness (JE) and job satisfaction (JS) were negatively associated with turnover intention (TI) (*B* = –0.331, *p* < 0.001; *B* = –0.343, *p* < 0.001). The interaction between JE and positive career shocks (PCS) was significant (*B* = –0.162, *p* < 0.01), indicating that PCS moderated the relationship between JE and TI. Similarly, the interaction between JS and PCS was significant (*B* = 0.225, *p* < 0.001), suggesting that PCS moderated the relationship between JS and TI.

**FIGURE 4:**
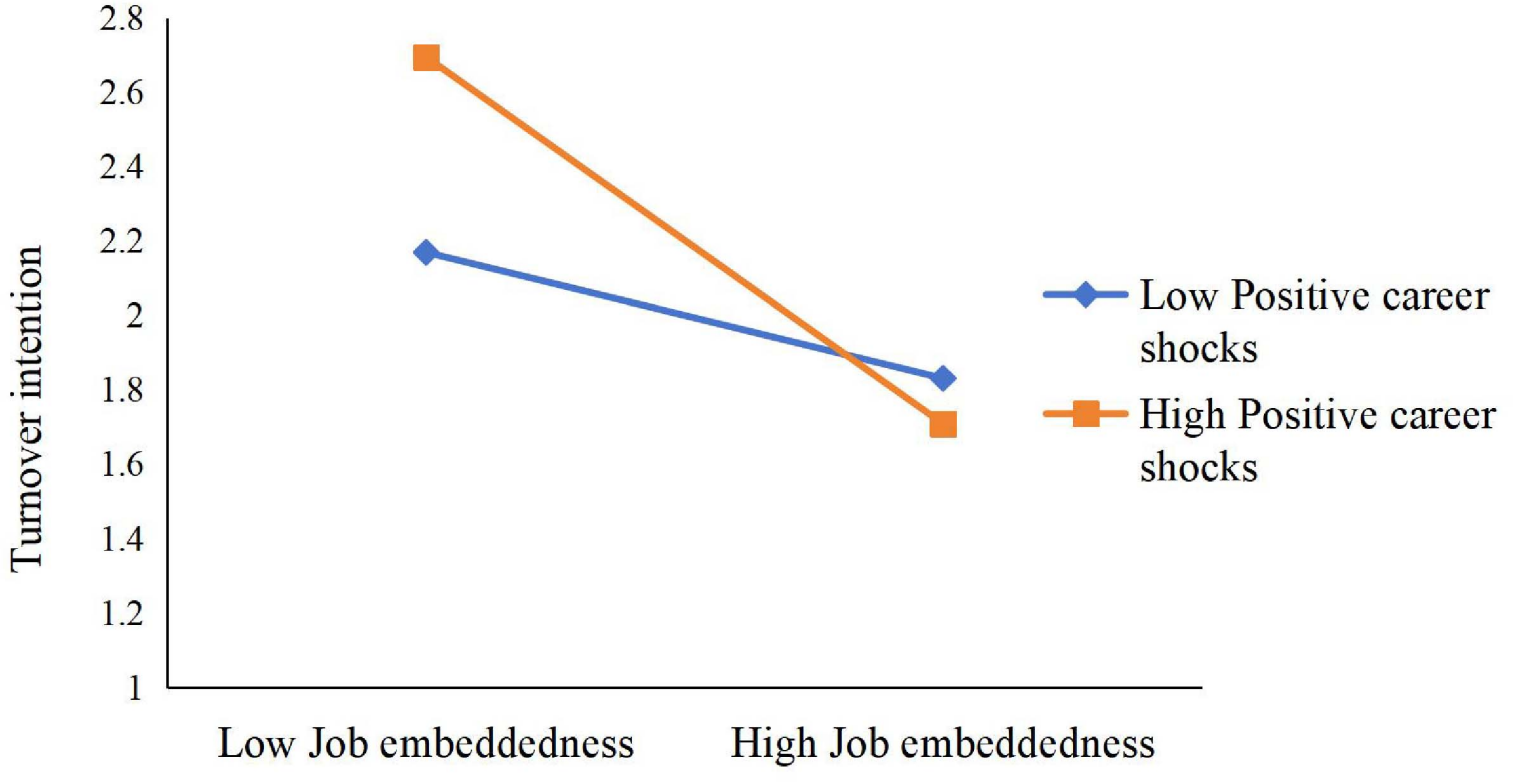
Moderation of positive career shocks to job embeddedness and turnover intention.

**FIGURE 5:**
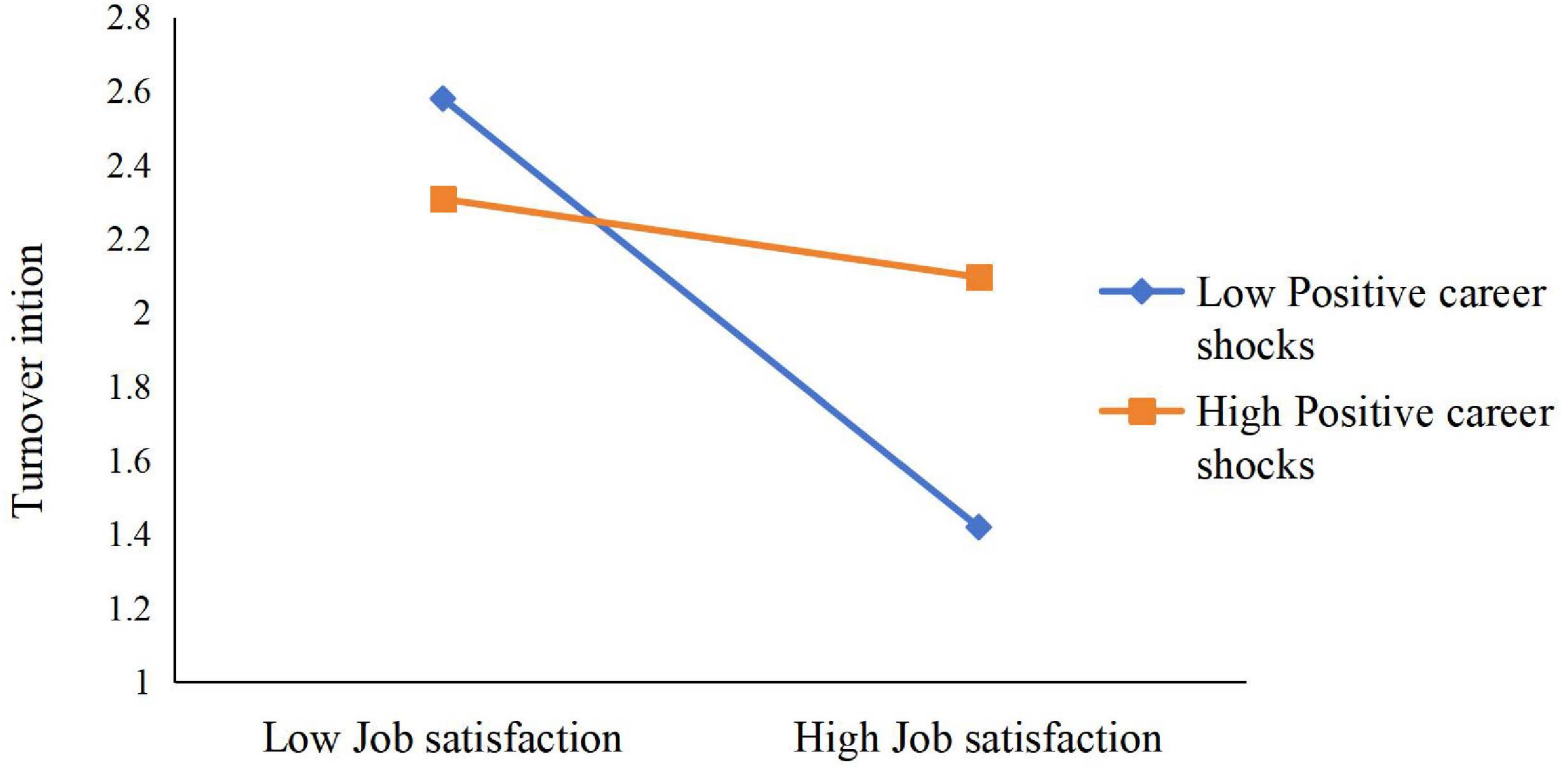
Moderation of positive career shocks to job satisfaction and turnover intention.

Simple slope analysis (see Figure 4) showed that when PCS was high, JE was negatively related to TI (B_simple_ = –0.5072, *p* < 0.001), but this effect was not significant when PCS was low (B_simple_ = –0.1539, n.s.). Likewise, when PCS was low (see Figure 5), JS was negatively related to TI (B_simple_ = –0.6011, *p* < 0.001), but this effect was not significant when PCS was high (B_simple_ = –0.0848, n.s.).

Negative career shocks (NCS) were positively associated with turnover intention (TI) (*B* = 0.148, *p* < 0.01). The interaction between leader-member exchange (LMX) and NCS was significant (*B* = –0.181, *p* < 0.01), indicating that NCS moderated the relationship between LMX and TI. Similarly, the interaction between job satisfaction (JS) and NCS was significant (*B* = 0.189, *p* < 0.01), indicating that NCS also moderated the relationship between JS and TI.

**FIGURE 6:**
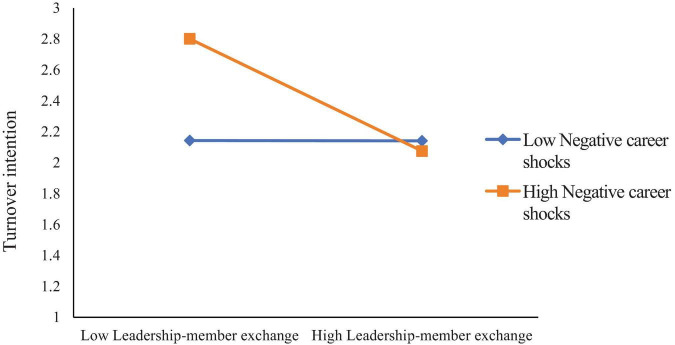
Moderation of negative career shocks to leadership-member exchange and turnover intention.

**FIGURE 7:**
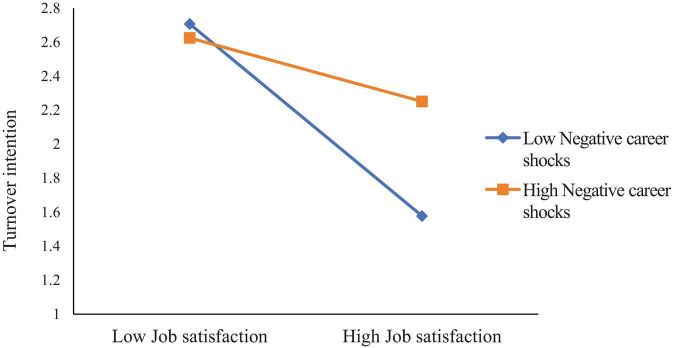
Moderation of negative career shocks to job satisfaction and turnover intention.

Simple slope tests (see Figure 6) showed that when NCS was high, LMX was negatively related to TI (B_simple_ = –0.3788, *p* < 0.001), but this effect was not significant when NCS was low (B_simple_ = 0.0152, n.s.). Likewise, when NCS was high (see Figure 7), the effect of JS on TI was not significant (B_simple_ = –0.1697, n.s.), but when NCS was low, JS was negatively related to TI (B_simple_ = –0.5818, *p* < 0.001).

Finally, the study’s findings revealed significant relationships between job embeddedness (JE) and job satisfaction (JS) with turnover intention (TI) (*B* = –0.316, *p* < 0.001; *B* = –0.292, *p* < 0.001). The study also highlighted the significant moderating role of negative career shocks (NCS) in the JE–TI and JS–TI relationships, underscoring the importance of considering NCS in organizational behavior research.

**FIGURE 8:**
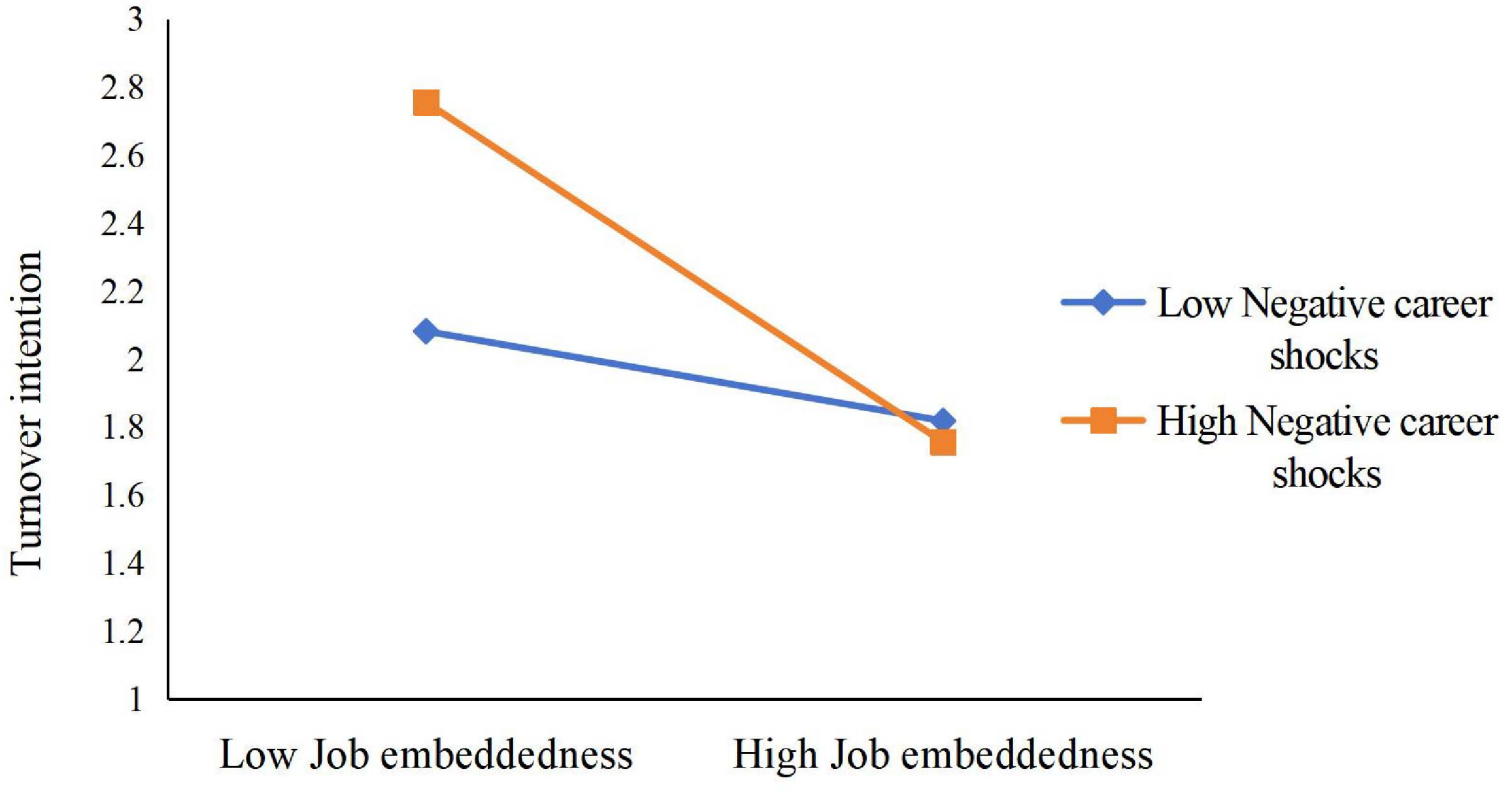
Moderation of negative career shocks to job embeddedness and turnover intention.

**FIGURE 9:**
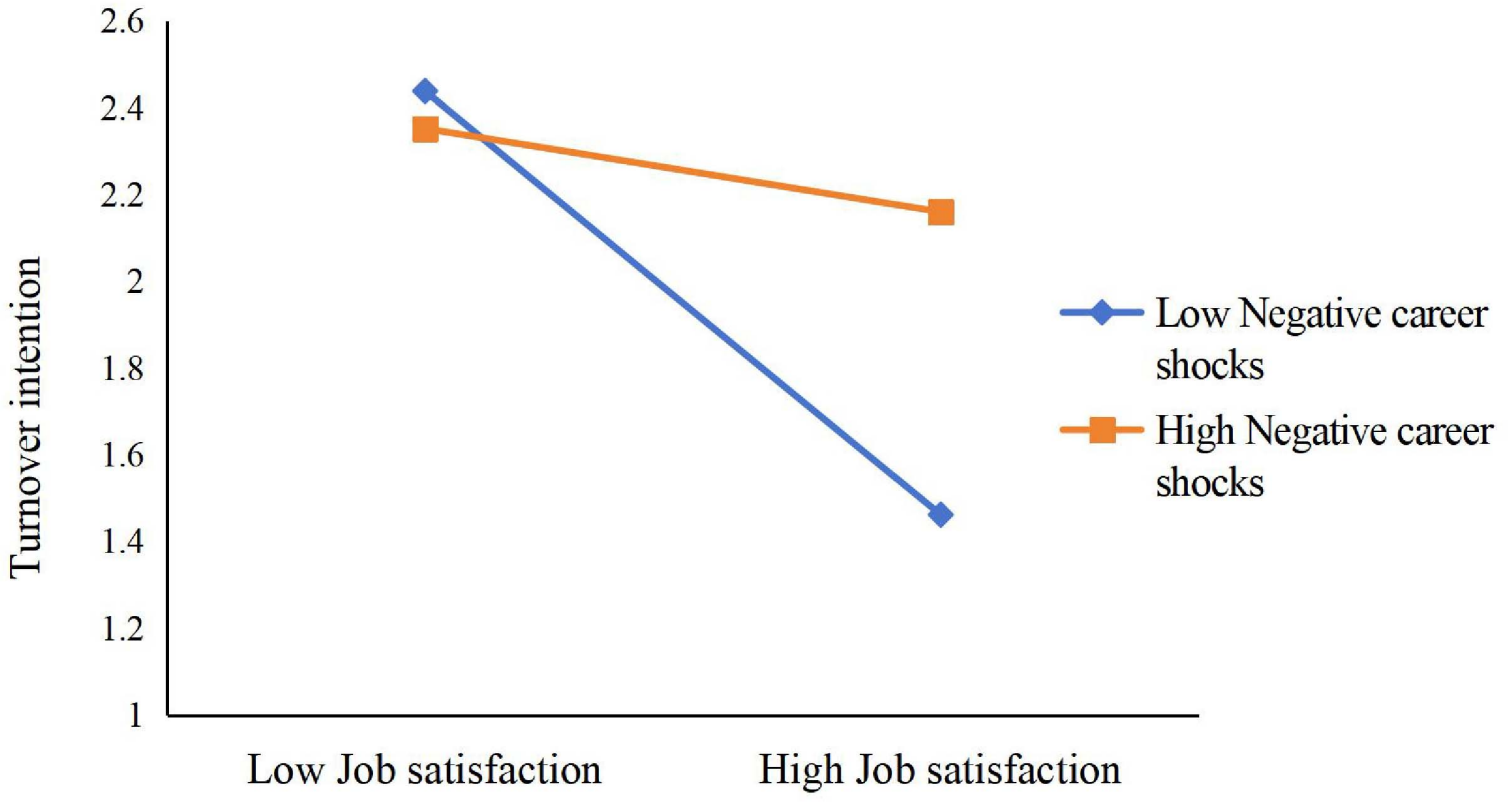
Moderation of negative career shocks to job satisfaction and turnover intention.

Simple slope analysis (see Figure 8) showed that when NCS was high, JE was negatively related to TI (B_simple_ = –0.5175, *p* < 0.001), but this effect was not significant when NCS was low (B_simple_ = –0.1152, n.s.). Likewise, when NCS was high (see Figure 9), JS was not significantly related to TI (B_simple_ = –0.0782, n.s.), but when NCS was low, JS was negatively related to TI (B_simple_ = –0.5065, *p* < 0.001).

## 5 Discussion

This study delves into the complex interplay of leader-member exchange (LMX), job embeddedness, job satisfaction, and career shocks and their impact on hospital staff’s turnover intention. The results reveal a significant negative LMX-TI link, in line with most previous studies (Le et al., 1993; Major et al., 1995; Sparrowe, 1994; Vecchio and Gobdel, 1984; Wilhelm et al., 1993), and confirm the role of high-quality LMX in reducing turnover intention. However, the proposed curvilinear LMX-TI relationship by Harris et al. (2005) was not supported in this study, possibly due to the high job stress and limited promotion chances among public hospital staff. These findings contribute to the existing body of knowledge, providing a deeper understanding of the factors influencing turnover intention in the healthcare sector.

The negative correlation between job embeddedness (JE) and turnover intention (TI) is consistent with the findings of Jiang et al. (2012), Lee et al. (2004), and Mitchell et al. (2001), indicating that employees’ deep integration into the organization significantly reduces their likelihood of leaving. This sense of integration primarily stems from employees’ identification with the organization, job satisfaction, and positive relationships with colleagues and superiors. To enhance employees’ organizational identification, managers can foster a strong company culture and provide opportunities for employees to contribute to the organization’s mission. To improve job satisfaction, managers can ensure fair compensation, provide opportunities for career growth, and promote a healthy work-life balance. These insights suggest practical strategies that managers can implement to improve their job embeddedness.

Job satisfaction (JS) emerges as a key player, fully mediating the relationship between leader-member exchange (LMX) and turnover intention. This finding, in line with the results of Han and Jekel (2011), further solidifies JS’s mediating role in the LMX-TI relationship. Additionally, JS was found to partially mediate the relationship between JE and TI, highlighting the direct impact of JE on employees’ turnover intention and its indirect effect through improved job satisfaction. This complexity underscores the need for a comprehensive approach to understanding and addressing turnover intention, engaging us in a committed exploration of this critical issue.

The moderating role of career shocks indicates that positive career shocks (e.g., promotions or project successes) can enhance employees’ job embeddedness and satisfaction, thereby reducing turnover intention. Conversely, negative career shocks (e.g., promotion failures or unfair treatment) may weaken employees’ trust in leaders and commitment to the organization, increasing the likelihood of leaving. This finding empirically supports career shocks theory and underscores the need for managers to understand and address employees’ psychological responses when they face career shocks, fostering a culture of empathy and consideration.

The limitations of this study are as follows. First, the study focused on medical staff in Guangzhou’s public hospitals, which may restrict the generalizability of the findings. Future research could expand to other industries or regions to verify the broad applicability of the results. Second, the two-wave survey design may be subject to random influences. Future research could increase the frequency of data collection to enhance data stability and reliability. Third, the sole use of quantitative methods limits an in-depth understanding of the subject’s behaviors, attitudes, and views. Future research could incorporate qualitative methods to complement the limitations of quantitative data and provide more nuanced information. It is also important for future research to explore the differentiated impacts of various positive and negative career shocks on turnover intention and how individual differences, such as career adaptability, moderate these effects, as this will provide a more nuanced understanding of these effects.

## 6 Conclusion

This study is centered on the roles of leader-member exchange (LMX), job embeddedness, job satisfaction, and career shocks in influencing hospital staff’s turnover intention. The results demonstrate a significant negative association between LMX and turnover intention, with job embeddedness and satisfaction serving as mediators in this relationship. Positive career shocks bolster job embeddedness and satisfaction, while negative shocks may diminish organizational commitment and increase the likelihood of departure. These findings underscore the pivotal roles of LMX, job embeddedness, employee retention satisfaction, and the moderating impact of career shocks in the context of hospital staff’s turnover intention.

Theoretically, this study introduces the moderating role of career shocks, offering a new perspective on how leader-member exchange (LMX) and job embeddedness influence employees’ turnover intention through psychological mechanisms. This extends the application of LMX and career shocks theories, providing new research directions for understanding occupational behaviors. Importantly, it offers actionable insights for public hospital managers, empowering them to effectively reduce turnover intention by enhancing LMX quality and boosting employees’ job embeddedness and satisfaction. Moreover, managers should monitor employees’ psychological responses to career shocks and offer timely support to mitigate the negative impacts of such shocks on turnover intention.

## Data Availability

The original contributions presented in the study are included in the article/supplementary material, further inquiries can be directed to the corresponding author.

## Footnotes

1
https://www.wjx.cn

## References

[B1] Akkermans J. Seibert S. E. Mol S. T. (2018). Tales of the unexpected: Integrating career shocks in the contemporary careers literature. *SA J. Ind. Psychol.* 44:a1503. 10.4102/sajip.v44i0.1503

[B2] Ali Z. Ghani U. Islam Z. U. Mehreen A. (2020). Measuring career shocks: A study of scale development and validation in the chinese context. *Aus. J. Career Dev.* 29 164–172. 10.1177/1038416220950737

[B3] Amarneh S. Raza A. Matloob S. Alharbi R. K. Abbasi M. A. (2021). The influence of person-environment fit on the turnover intention of nurses in jordan: The moderating effect of psychological empowerment. *Nurs. Res. Pract.* 2021:6688603. 10.1155/2021/6688603 33815841 PMC7987446

[B4] Chen L. Y. (2021). Analysis on the current situation and countermeasures of brain drain in tertiary public hospitals. *Jiangsu Health Syst. Manag.* 32 1025–1028.

[B5] Crossley C. D. Bennett R. J. Jex S. M. Burnfield J. L. (2007). Development of a global measure of job embeddedness and integration into a traditional model of voluntary turnover. *J. Appl. Psychol.* 92 1031–1042. 10.1037/0021-9010.92.4.1031 17638463

[B6] Deng Y. (2023). Correlation between career transition shock and perceived organizational support and empathy ability of newly recruited nurses in oncology department. *Tianjin J. Nurs.* 31 656–659. 10.3969/j.issn.1006-9143.2023.06.006

[B7] Dong A. Q. Tong X. L. Yang Z. X. Wang Q. W. Zhang X. (2024). Research on the current situation of new nurses’ transformation impact and its correlation with turnover intention. *Occup. Health* 40 3362–3365. 10.13329/j.cnki.zyyjk.2024.0620

[B8] Fang Y. C. Chen J. Y. Zhang X. D. Dai X. X. Tsai F. S. (2020). The impact of inclusive talent development model on turnover intention of new generation employees: The mediation of work passion. *Int. J. Environ. Res. Public Health* 17:6054. 10.3390/ijerph17176054 32825300 PMC7503779

[B9] Feng J. Jiang X. L. Zhou W. X. (2021). Career shocks: Conceptualizations, measurements, antecedents and consequences. *Hum. Resources Dev. China* 38 6–24. 10.16471/j.cnki.11-2822/c.2021.5.001

[B10] Gerstner C. R. Day D. V. (1997). Meta-Analytic review of leader–member exchange theory: Correlates and construct issues. *J. Appl. Psychol.* 82 827–844. 10.1037/0021-9010.82.6.827

[B11] Graen G. B. Uhl-Bien M. (1995). Relationship-based approach to leadership: Development of leader-member exchange (LMX) theory of leadership over 25 years: Applying a multi-level multi-domain perspective. *Leadersh. Quar.* 6 219–247. 10.1016/1048-9843(95)90036-5

[B12] Griffeth R. W. Hom P. W. Gaertner S. (2000). Meta-analysis of antecedents and correlates of employee turnover: Update, moderator tests, and research implications for the next millennium. *J. Manag.* 26 463–488. 10.1177/014920630002600305

[B13] Han G. H. Jekel M. (2011). The mediating role of job satisfaction between leader-member exchange and turnover intentions. *J. Nurs. Manag.* 19 41–49. 10.1111/j.1365-2834.2010.01184.x 21223404

[B14] Harris K. J. Kacmar K. M. Witt L. A. (2005). An examination of the curvilinear relationship between. leader-member exchange and intent to turnover. *J. Organ. Behav.* 26 363–378. 10.1002/job.314

[B15] Hom P. W. Griffeth R. W. (1995). *Employee turnover.* Nashville, TN: Southwestern College Publishing.

[B16] Hom P. W. Lee T. W. Shaw J. D. Hausknecht J. P. (2017). One hundred years of employee turnover theory and research. *J. Appl. Psychol.* 102 530–545. 10.1037/apl0000103 28125259

[B17] Huang L. Cui Y. (2021). “Research review and prospects of career shocks,” in *Nanjing Bus Review*, ed. Liu Z. B. (Beijing: Economy & Management Publishing House), 184–194.

[B18] Jackofsky E. F. Peters L. H. (1983). Job turnover versus company turnover: Reassessment of the March and Simon participation hypothesis. *J. Appl. Psychol.* 68 490–495. 10.1037/0021-9010.68.3.490

[B19] Jiang K. Liu D. McKay P. F. Lee T. W. Mitchell T. R. (2012). When and how is job embeddedness predictive of turnover? A meta-analytic investigation. *J. Appl. Psychol.* 97 1077–1096. 10.1037/a0028610 22663557

[B20] Judge T. A. Weiss H. M. Kammeyer-Mueller J. D. Hulin C. L. (2017). Job attitudes, job satisfaction, and job affect: A century of continuity and of change. *J. Appl. Psychol.* 102 356–374. 10.1037/apl0000181 28125260

[B21] Kraimer M. L. Greco L. Seibert S. E. Sargent L. D. (2019). An investigation of academic career success: The new tempo of academic life. *AMLE* 18 128–152. 10.5465/amle.2017.0391

[B22] Le B. P. M. de J. R. D. Geersing J. Furda J. Komproe I. H. (1993). Leader member exchanges: Distinction between two factors. *Eur. Work Organ. Psychol.* 3 297–309. 10.1080/09602009308408599

[B23] Lee T. H. Gerhart B. Weller I. Trevor C. O. (2008). Understanding voluntary turnover: Path-specific job satisfaction effects and the importance of unsolicited job offers. *AMJ* 51 651–671. 10.5465/AMJ.2008.33665124

[B24] Lee T. W. Mitchell T. R. Sablynski C. Burton J. Holtom B. (2004). The effects of job embeddedness on organizational citizenship, job performance, volitional absences, and voluntary turnover. *Acad. Manag. J.* 47 711–722. 10.2307/20159613

[B25] Maertz C. P. Campion M. A. (2004). Profiles in quitting: Integrating process and content turnover theory. *Acad. Manag. J.* 47 566–582. 10.2307/20159602

[B26] Major D. A. Kozlowski S. W. J. Chao G. T. Gardner P. D. (1995). A longitudinal investigation of newcomer expectations, early socialization outcomes, and the moderating effects of role development factors. *J. Appl. Psychol.* 80 418–431. 10.1037/0021-9010.0.80.3.418

[B27] Mitchell T. R. Holtom B. C. Lee T. W. Sablynski C. J. Erez M. (2001). Why people stay: Using job embeddedness to predict voluntary turnover. *AMJ* 44 1102–1121. 10.2307/3069391

[B28] Mobley W. H. Griffeth R. W. Herbert H. H. Hand H. H. Hand H. H. Meglino B. M. (1979). Review and conceptual analysis of the employee turnover process. *Psychol. Bull.* 86 493–522. 10.1037/0033-2909.86.3.493

[B29] Mobley W. H. Horner S. O. Hollingsworth A. T. (1978). An evaluation of precursors of hospital employee turnover. *J. Appl. Psychol.* 63 408–414. 10.1037/0021-9010.63.4.408 701211

[B30] National Health Commission (2021). *China health statistical yearbook 2021.* New Delhi: National Health Commission.

[B31] Podsakoff P. M. MacKenzie S. B. Lee J. Y. Podsakoff N. P. (2003). Common method biases in behavioral research: A critical review of the literature and recommended remedies. *J. Appl. Psychol.* 88 879–903. 10.1037/0021-9010.88.5.879 14516251

[B32] Seibert S. E. Kraimer M. L. Heslin P. A. (2016). Developing career resilience and adaptability. *Organ. Dynam.* 45 245–257. 10.1016/j.orgdyn.2016.07.009

[B33] Seibert S. E. Kraimer M. L. Holtom B. C. Pierotti A. J. (2013). Even the best laid plans sometimes go askew: Career self-management processes, career shocks, and the decision to pursue graduate education. *J. Appl. Psychol.* 98 169–182. 10.1037/a0030882 23181345

[B34] Setthakorn K. P. Rostiani R. Schreier C. (2024). A meta-analytic review of job embeddedness and turnover intention: Evidence from South-East Asia. *Sage Open* 14:21582440241260092. 10.1177/21582440241260092

[B35] Sparrowe R. T. (1994). Empowerment in the hospitality industry: An exploration of antecedents and outcomes. *Hospital. Res. J.* 17 51–73. 10.1177/109634809401700306

[B36] Tsui A. S. Egan T. D. III O’Reilly C. A. (1992). Being different: Relational demography and organizational attachment. *Admin. Sci. Quar.* 37 549–579. 10.2307/2393472

[B37] Vecchio R. P. Gobdel B. C. (1984). The vertical dyad linkage model of leadership: Problems and prospects. *Organ. Behav. Hum. Perform.* 34 5–20. 10.1016/0030-5073(84)90035-7

[B38] Wang H. Law K. S. Hackett R. D. Wang D. X. Chen Z. X. (2005). Leader-member exchange as a mediator of the relationship between transformational leadership and followers’ performance and organizational citizenship behavior. *Acad. Manag. J.* 48 420–432. 10.5465/amj.2005.17407908

[B39] Wang N. (2021). Research on the causes and countermeasures of the current situation of brain drain in the medical system. *Smart Healthcare* 7 174–176.

[B40] Wilhelm C. C. Herd A. M. Steiner D. D. (1993). Attributional conflict between managers and subordinates: An investigation of leader-member exchange effects. *J. Organ. Behav.* 14 531–544. 10.1002/job.4030140603

[B41] Wu L. D. Dou L. Sun Q. Sun X. J. (2021). Analysis of the influence of workplace social capital on the turnover intention of medical staff in public hospitals. *Chin. Hospit. Manag.* 41 81–84.

[B42] YIMI Research (2022). Available online at: https://www.yxj.org.cn/detailPage?articleId=333394 (accessed April 6, 2025).

